# Lipid alterations play a role in the integration of PD-1/PD-L1 inhibitors and anlotinib for the treatment of advanced non–small-cell lung cancer

**DOI:** 10.1186/s12944-023-01960-7

**Published:** 2024-01-13

**Authors:** Li Liu, Shuo Zhang, Hai-Yan Yang, Chun-Hua Zhou, Yi Xiong, Nong Yang, Ye Tian

**Affiliations:** 1 The Second Affiliated Hospital of Soochow University, Suzhou, China; 2 Department of Medical Oncology, Lung Cancer and Gastrointestinal Unit, Hunan Cancer Hospital/The Affiliated Cancer Hospital of Xiangya School of Medicine, Central South University, Changsha, 410013 China; 3 Zhu Zhou Central Hospital, Zhuzhou, 412007 China

**Keywords:** Advanced NSCLC, Lipid metabolism, Anlotinib alongside PD-1/PD-L1 inhibitors, Therapeutic effect

## Abstract

**Background:**

Studies have shown that integrating anlotinib with programmed death 1 (PD-1)/programmed death-ligand 1 (PD-L1) inhibitors enhances survival rates among progressive non–small-cell lung cancer (NSCLC) patients lacking driver mutations. However, not all individuals experience clinical benefits from this therapy. As a result, it is critical to investigate the factors that contribute to the inconsistent response of patients. Recent investigations have emphasized the importance of lipid metabolic reprogramming in the development and progression of NSCLC.

**Methods:**

The objective of this investigation was to examine the correlation between lipid variations and observed treatment outcomes in advanced NSCLC patients who were administered PD-1/PD-L1 inhibitors alongside anlotinib. A cohort composed of 30 individuals diagnosed with advanced NSCLC without any driver mutations was divided into three distinct groups based on the clinical response to the combination treatment, namely, a group exhibiting partial responses, a group manifesting progressive disease, and a group demonstrating stable disease. The lipid composition of patients in these groups was assessed both before and after treatment.

**Results:**

Significant differences in lipid composition among the three groups were observed. Further analysis revealed 19 differential lipids, including 2 phosphatidylglycerols and 17 phosphoinositides.

**Conclusion:**

This preliminary study aimed to explore the specific impact of anlotinib in combination with PD-1/PD-L1 inhibitors on lipid metabolism in patients with advanced NSCLC. By investigating the effects of using both anlotinib and PD-1/PD-L1 inhibitors, this study enhances our understanding of lipid metabolism in lung cancer treatment. The findings from this research provide valuable insights into potential therapeutic approaches and the identification of new therapeutic biomarkers.

**Supplementary Information:**

The online version contains supplementary material available at 10.1186/s12944-023-01960-7.

## Introduction

Lung malignancy stands as the foremost contributor to sickness and demise linked to neoplasms. Non–small-cell lung cancer (NSCLC) is the most common variant and exhibits a disheartening outlook; this is primarily due to the frequent occurrence of locally advanced or disseminated metastasis in the majority of patients upon initial diagnosis or after surgical intervention [1]. Classical chemotherapy exhibits restricted efficacy in the management of NSCLC, with a wide range of overall response rates varying from 6.7 to 10.8%, and a meager 5-year survival rate ranging from 7 to 14% [2]. However, there have been significant changes in the treatment landscape for NSCLC in recent years primarily as a result of the introduction of immunotherapy [3]. The field of immunotherapy has recently brought about a revolutionary shift in the treatment of NSCLC across diverse scenarios, thus playing a vital role in augmenting the well-being of these individuals [4].

Numerous clinical studies have consistently demonstrated the effectiveness of immune-checkpoint inhibitors (ICIs) in the treatment of diverse conditions. Evidence has corroborated the efficacy of anti-programmed death 1 (PD-1) antibodies, anti–PD-1 ligand (PD-L1) antibodies, and anti–cytotoxic T-lymphocyte–associated protein 4 (CTLA-4) antibodies [5]. Nonetheless, most patients with NSCLC do not note substantial advantages solely from immunotherapy [6]; therefore, it is essential to investigate the potential of combination therapies to enhance the efficacy of immunotherapy.

Anlotinib, a multi-targeted anti-angiogenic agent, is a small-molecule compound that has been shown to have inhibitory effects on both tumor cells and angiogenesis [7]. New findings from recent scientific research have provided strong evidence suggesting that the combination of anlotinib and PD-1 inhibitors could boost results for individuals with advanced lung cancer, specifically improving both progression-free survival (PFS) and overall survival (OS) [8]. Despite positive results observed in patients with NSCLC with negative driver mutations, there are still some individuals who do not benefit from this treatment. The precise factors contributing to this lack of response have not yet been elucidated [9, 10].

Lipids have a vital function as structural constituents of cellular membranes and as secondary messengers within cells. Emerging data have progressively underscored the noteworthy involvement of lipids in the development of diverse forms of cancer, such as lung cancer [11–13]. Long-chain fatty acyl-CoA synthetases (ACSLs), which have been discovered to have the potential to promote the upregulation of lipids, are recognized for their significant role in breast and colorectal cancer and their possible oncogenic properties. However, interestingly, they also exhibit potential tumor-suppressor properties in lung cancer [14]. Increased levels of lipids, including phospholipids, neutral lipids, and triglycerides, have been observed in lung cancer [15]. Furthermore, alterations in sphingolipid metabolism have also been identified in lung cancer. The presence of sphingosine kinase 2 (SPHK2) has been linked to unfavorable survival outcomes in NSCLC as well as resistance to gefitinib EGFR TKI therapy [16]. In general, the reprogramming of lipid metabolism has become a crucial contributor to the advancement and progression of lung cancer.

As described in this article, our research revealed that patients with advanced NSCLC lacking driver mutations displayed distinct responses upon receiving a combination therapy involving a PD-1 inhibitor and anlotinib. Further, a comparative analysis of lipid composition in patients who underwent treatment with anlotinib in conjunction with PD-1/PD-L1 inhibitors was conducted. The findings demonstrated that, in the group showing partial responses(PR), there were no notable alterations in lipids between before and after treatment. However, in the group with stable disease (SD), only one phosphatidylglycerol (PG) and three phosphatidylinositol (PIs) exhibited a significant increase after therapy. Conversely, among patients with progressive disease (PD), there was a substantial upregulation in two PGs and 17 PIs. The aforementioned results suggest that ensuring a well-balanced lipid profile is crucial for effectively treating patients with advanced NSCLC lacking driver mutations. This can be achieved by employing the combination of anlotinib and PD-1/PD-L1 inhibitors. Notably, an elevation in PG levels—specifically, the level of PI—after treatment may result in an unfavorable treatment response. By broadening the comprehension of lipid metabolism in lung cancer, this investigation enhances the understanding of potential therapeutic strategies and facilitates the discovery of novel therapeutic biomarkers.

## Materials and methods

### Participants

Clinical records were collected from patients with advanced NSCLC with negative driver mutations at Hunan Cancer Hospital between July 2018 and March 2022. In the case of adenocarcinoma patients, it was recommended to utilize tissue samples for NGS sequencing. Patients without EGFR/ALK/ROS-1 driver mutations were included in the analysis. However, according to the 2022 Chinese Society of Clinical Oncology (CSCO) Guidelines for the Diagnosis and Treatment of non-small cell lung Cancer, genetic testing is not recommended for patients with advanced squamous cell carcinoma due to the extremely low EGFR/ALK/ROS-1 mutation ratio. The guidelines suggest that patients with lung squamous cell carcinoma, who can be considered genetically negative drivers, should receive conventional anti-tumor therapy. In this study, 7 out of the 15 recruited lung squamous cell carcinoma patients voluntarily underwent genetic testing, and the results showed that they tested negative for EGFR/ALK/ROS-1 mutations. This finding further supports the observation of a low mutation rate of these genes in lung squamous cell carcinoma. Therefore, the remaining 8 advanced lung squamous cell carcinoma patients who did not undergo genetic testing can also be considered as negative gene drive patients based on CSCO guidelines. The enrolled 30 participants who underwent a treatment regimen consisting of the administration of chemotherapy in conjunction with ICIs, either as their initial or subsequent therapeutic approach. After, when resistance to chemotherapy and ICIs emerged, the patients were given ICIs in combination with anlotinib. Blood samples were taken from enrolled patients before and after treatment involving ICIs and anlotinib. The clinical stage of each patient was determined using the eighth edition of the TNM classification. Prior to the administration of ICIs and anlotinib, the Eastern Cooperative Oncology Group (ECOG) guideline was used to evaluate the performance status. To qualify for inclusion in the present investigation, individuals needed to: (i) exhibit an ECOG performance status ranging from 0 to 1 as well as a histologically confirmed NSCLC clinical stage IIIb–IIIc or IV, (ii) have completed at least two courses of ICI plus anlotinib therapy, (iii) have evaluable disease, and (iv) not have any organ dysfunction. Participants with severe autoimmune diseases or those requiring systemic treatment with corticosteroids or other immunosuppressive medications were excluded from the study. The ethical approval document for the study (no. SBQLL-2021-092) was granted by the Hunan Cancer Hospital. All of the enrolled individuals provided informed consent by signing consent forms prior to their participation in the experiment.

### Therapy

Patients with advanced NSCLC who experienced progression after receiving at least one round of chemotherapy as well as ICIs were subjected to a re-challenge involving the combination of ICIs and anlotinib. For a total of 14 days, patients were administered anlotinib orally at a dosage of 12 mg per day, which was followed by a one-week pause in the regimen. In a 21-day cycle, patients were administered a PD-1 inhibitor via intravenous injection on the first day, such as toripalimab (240 mg), carrelizumab (200 mg), sintilimab (200 mg), or pembrolizumab (200 mg). The treatment was continued until any of the following conditions occurred: progressive disease or death, patient refusal, unacceptable toxicity, pregnancy, or treatment withdrawal for any other reason. The evaluation of the response was conducted based on the Response Evaluation Criteria in Solid Tumors (RECIST) version 1.1, using enhanced computed tomography (CT) scans recorded at two-month intervals [17].

### Lipidomic analysis

A revised approach inspired by the methodology outlined in Xuan et al.‘s study was used to analyze the lipid samples [18]. In brief, venous blood was collected into tubes containing heparin to prevent coagulation. The blood was then centrifuged using 2000 g lasting 15 min under 4 °C to obtain the serum component. The serum was mixed with 80 µL of methanol and 400 µL of MTBE (tert-butyl methyl ether). To obtain the serum component, the blood was centrifuged under 2000 g for 15 min at a temperature of 4 °C. Next, the serum was combined with 80 µL of methanol as well as 400 µL of MTBE (tert-butyl methyl ether), along with lipid standards.

This mixture was vigorously vortexed for 30 s and then subjected to centrifugation to separate the upper phase. The separated phases were carefully collected and dried using vacuum evaporation. Finally, the desiccated samples were reconstituted by utilizing 100 µL of a blend consisting of methanol and methylene chloride in an equal proportion of 1:1.

For lipid analysis, a mass spectrometer (QTRAP 6500; Danaher Corporation, Toronto, Canada) coupled by a Shimadzu LC-30 A (Shimadzu, Japan) system was used. To separate the lipid components, a CQUITY UPLC® BEH C18 column (2.1 × 100 mm, 1.7 μm; Waters Corp., Milford, MA, USA) was employed. To ensure optimal chromatographic performance, the following set of conditions was established: the oven temperature was maintained for 55 °C, 0.26 mL/min was established as flow rate, along with an injection volume of 5 µl. The mobile phase was composed of two solutions: solution A (a mixture of H_2_O and acetonitrile in a 40:60 ratio, v/v, containing 10 mM of ammonium acetate) and solution B (a mixture of acetonitrile and isopropanol in a 10:90 ratio, v/v, containing 10 mM of ammonium acetate).

A gradient, consisting of varying proportions of two solutions (referred to as solutions A and B), was employed in this study. The mobile-phase composition during different time intervals was as follows: from 0 to1.5 min, 68% solution A and 32% solution B were used; from 1.5 to 15.5 min, the proportions of solution A and solution B were 15% and 85%, respectively; from 15.5 to 15.6 min, 3% solution A and 97% solution B were utilized; from 15.6 to 18 min, the same proportions of solution A and solution B were used as in the previous time interval; from 18 to 18.1 min, the mobile phase reverted back to 68%solution A and 32% solution B of; and, from 18.1 to 20 min, the same proportions of solution A and solution B were maintained. For the electrospray ionization, specific parameters were set. The curtain gas pressure was maintained at 20 psi, while the atomizing gas pressure was set at 60 psi. The ion source voltage was alternated between − 4500 and 5500 V, depending on the specific condition. The ion source temperature was carefully controlled and maintained at a constant 600 °C. In addition, an auxiliary gas pressure of 60 psi was applied. Multiple reaction monitoring was employed to monitor the reactions and obtain precise data. Moreover, quality control samples were incorporated to ensure the accuracy and reliability of the liquid chromatography-mass spectrometry examination. These quality control samples were created by blending samples under the same test conditions, and they were analyzed every third sample in order to evaluate the overall quality of the data.

### Statistical analysis

The measurement of peak area determined the abundance of lipids in this study. After, the obtained data were processed and normalized using the website https://www.metaboanalyst.ca/. This online platform is primarily focused on processing raw spectra, conducting general statistical analysis, and performing functional analysis [19, 20]. In order to evaluate the highest covariance between lipidomic samples, i.e., both those treated with a PD-1 inhibitor and anlotinib, and those left untreated, the researchers conducted the discriminant analysis of partial least squares (PLS-DA). Examination of lipid molecule correlations was conducted using correlation heatmaps. The mean ± standard error of the mean format was used to present the data. A paired two-tailed Student’s *t* test was carried out to analyze the comparison between the non-treatment group and the conjunction of anlotinib and a PD-1 inhibitor–treated group. *P* < 0.05 was considered statistical significance.

## Results

### Demographic information and therapeutic effect among patients

A total of 30 advanced NSCLC patients lacking driver mutations were enrolled in this study. Tables 1 and 2 present the demographic details of these patients. As outlined in the methods section, the patients received the specified treatment regimen. Based on the combined effects of anlotinib and PD-1/PD-L1 inhibitors, patients were stratified into three groups. The first group (*n* = 6) showed partial remission of the tumor after therapy (PR), the second group (*n* = 17) had a tumor that remained stable after therapy (SD), and the third group (*n* = 7) experienced tumor progression after therapy (PD).

**Table 1:** Patients characteristics

Characteristic	ICIs plus anlotinib No (%)
No of total patients	30
**Gender**	
Male	25(83.33%)
Female	5(16.67%)
**Age(years)**	
Median age(range)	63.5(range,36–79)
<65	16(53.33%)
≥ 65	14(46.67%)
**Smoking History**	
Non-smoker	5(16.67%)
Former smoker	25(83.33%)
**ECOG performance status**	
0	10(33.33%)
1	20(66.67%)
**Histology**	
Squamous cell carcinoma	15(50.00%)
Adenocarcinoma	15(50.00%)
**Stage**	
IIIb-IIIc	5(16.67%)
IV	25(83.33%)
**Metastatic**	
Liver	5(16.67%)
Brain	4(13.33%)
Bone	10(33.33%)
Lung	16(53.33%)
Pleural metastasis	4(13.33%)
**PD-L1 status test**	
Data unavailable	9(30.00%)
0%	5(16.67%)
1–49%	6(20.00%)
≥ 50%	10(33.33%)
**PFS**	
≥ 6个月	15(50.00%)
<6个月	15(50.00%)
**Efficacy evaluation**	
PR	6(20.00%)
SD	17(56.67%)
PD	7 (23.33%)

**Table 2:** Patients characteristics in each response group (PR, SD, and PD)

Efficacy evaluation		PR	SD	PD
No of total patients		6	17	7
**Gender**	Male	4(66.67%)	16(94.12%)	5(71.43%)
	Female	2(33.33%)	1(5.88%)	2(28.57%)
**Age(years)**	*P* = 0.8784 (PR vs. SD) *P* = 0.7409 (PR vs. PD) *P* = 0.3373 (SD vs. PD)	59.50 ± 9.73	62.17 ± 10.18	54.71 ± 13.91
**Height(cm)**	*P* = 0.1646 (PR vs. SD) *P* = 0.8785 (PR vs. PD) *P* = 0.1646 (SD vs. PD)	161.33 ± 9.11	165.47 ± 5.18	161.86 ± 5.15
**Weight(Kg)**	*P* = 0.8951 (PR vs. SD) *P* = 0.5347 (PR vs. PD) *P* = 0.8651 (SD vs. PD)	60.50 ± 15.24	59.76 ± 11.93	56.43 ± 5.99
**BMI(Kg/m**^**2**^**)**	*P* = 0.4892 (PR vs. SD) *P* = 0.5287 (PR vs. PD) *P* = 0.9610 (SD vs. PD)	22.97 ± 5.15	21.70 ± 3.65	21.61 ± 2.81
**Smoking History**	Non-smoker	2(33.33%)	2(11.76%)	1(14.29%)
	Former smoker	4(66.67%)	15(88.24%)	6(85.71%)
**ECOG performance status**	0	3(50.00%)	5(29.41%)	2(28.57%)
	1	3(50.00%)	12(70.59%)	5(71.43%)
**Histology**	Squamous cell carcinoma	3(50.00%)	7(41.18%)	5(71.43%)
	Adenocarcinoma	3(50.00%)	10(58.82%)	2(28.57%)
**Stage**	IIIb-IIIc	3(50.00%)	2(11.76%)	0(0.00%)
	IV	3(50.00%)	15(88.24%)	7(100.00%)
**Metastatic**	Liver	2(33.33%)	2(11.76%)	1(14.29%)
	Brain	1(16.67%)	2(11.76%)	1(14.29%)
	Bone	2(33.33%)	5(29.41%)	3(42.86%)
	Lung	2(33.33%)	10(58.82%)	4(57.14%)
	Pleural metastasis	0(0.00%)	3(17.65%)	1(14.29%)
**PD-L1 status test**	Data unavailable	3(50.00%)	3(17.65%)	3(42.86%)
	0%	1(16.67%)	2(11.76%)	2(28.57%)
	1–49%	1(16.67%)	3(17.65%)	2(28.57%)
	≥ 50%	1(16.66%)	9(52.94%)	0(0.00%)

### A lipid composition analysis was conducted on patients exhibiting advanced NSCLC who underwent a combination treatment involving PD-1/PD-L1 inhibitors and anlotinib

We conducted an analysis of the lipid profiles to investigate the factors contributing to the varying treatment outcomes among patients. The lipid components of all three of the patient groups before and after therapy were examined. Through lipidomic analysis, a total of 460 lipids were identified, which can be classified into 18 subclasses. The different categories encompass various subclasses of lipids—namely, phosphatidylethanolamine, phosphatidic acid, phosphatidylcholine, PI, PG, phosphatidylserine, lysophosphatidylethanolamine, lysophosphatidic acid, lysophosphatidylcholines, lysophosphatidylinositol, lysophosphatidylglycerol, triacylglycerol, diacylglycerol, cholesteryl ester, fatty acid, ceramide, hexosylceramide, and sphingomyelin.

**Fig. 1:**
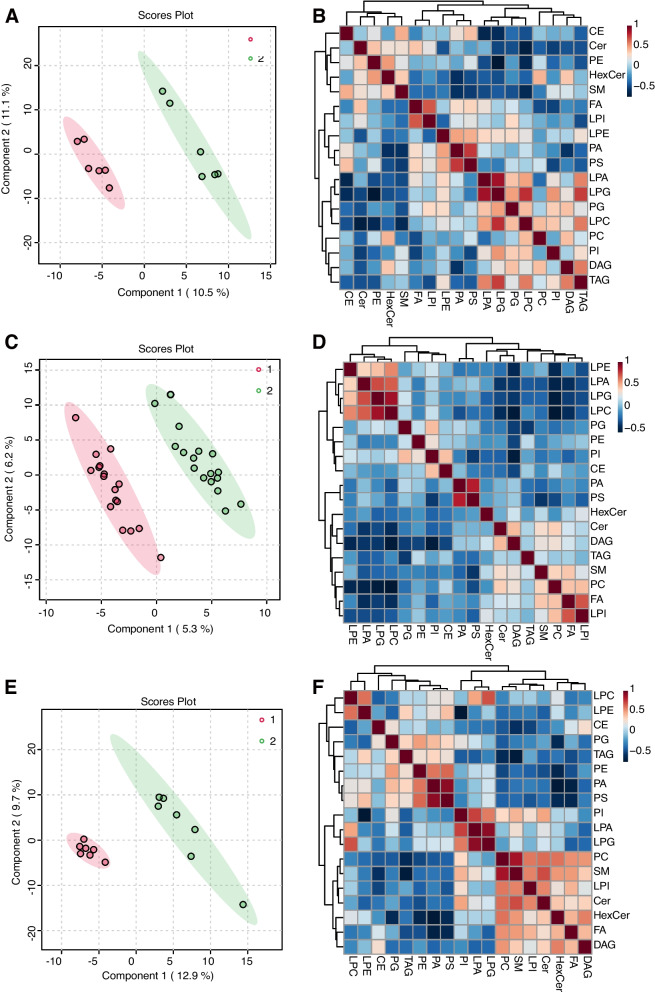
Lipidomic patterns in advanced NSCLC individuals pre and post administration of PD-1/PD-L1 inhibitors along with anlotinib. **A **The PLS-DA analysis was performed on advanced NSCLC patients in the PR group (*n* = 6). The non-treated PR group was represented by ‘1’ and the anlotinib along with PD-1/PD-L1 inhibitors-treated PR group was represented by ‘2’. **B** Correlation analysis was carried out on PR group subjects with advanced NSCLC to examine the associations among distinct lipids. Various colors were utilized to depict the correlation strength, employing the Pearson’s correlation coefficient. **C **The PLS-DA analysis was carried out on SD group subjects with advanced NSCLC patients (*n *= 17). The non-treated PR group was represented by ‘1’ and anlotinib along with PD-1/PD-L1 inhibitor -treated SD group was represented by ‘2’. **D **Correlation analysis was carried out on SD group subjects with advanced NSCLC to examine the associations among distinct lipids. Various colors were utilized to depict the correlation strength, employing the Pearson’s correlation coefficient. **E **The PLS-DA analysis was performed on advanced NSCLC patients in the PD group (*n *= 7). The non-treated PD group was represented by ‘1’ and anlotinib along with PD-1/PD-L1 inhibitor-treated PD group was represented by ‘2’. **F **Correlation analysis was performed on advanced NSCLC patients in the PD group to investigate the significantly diverse lipids. The level of Pearson’s correlation coefficient was represented using various colors

**Fig. 2:**
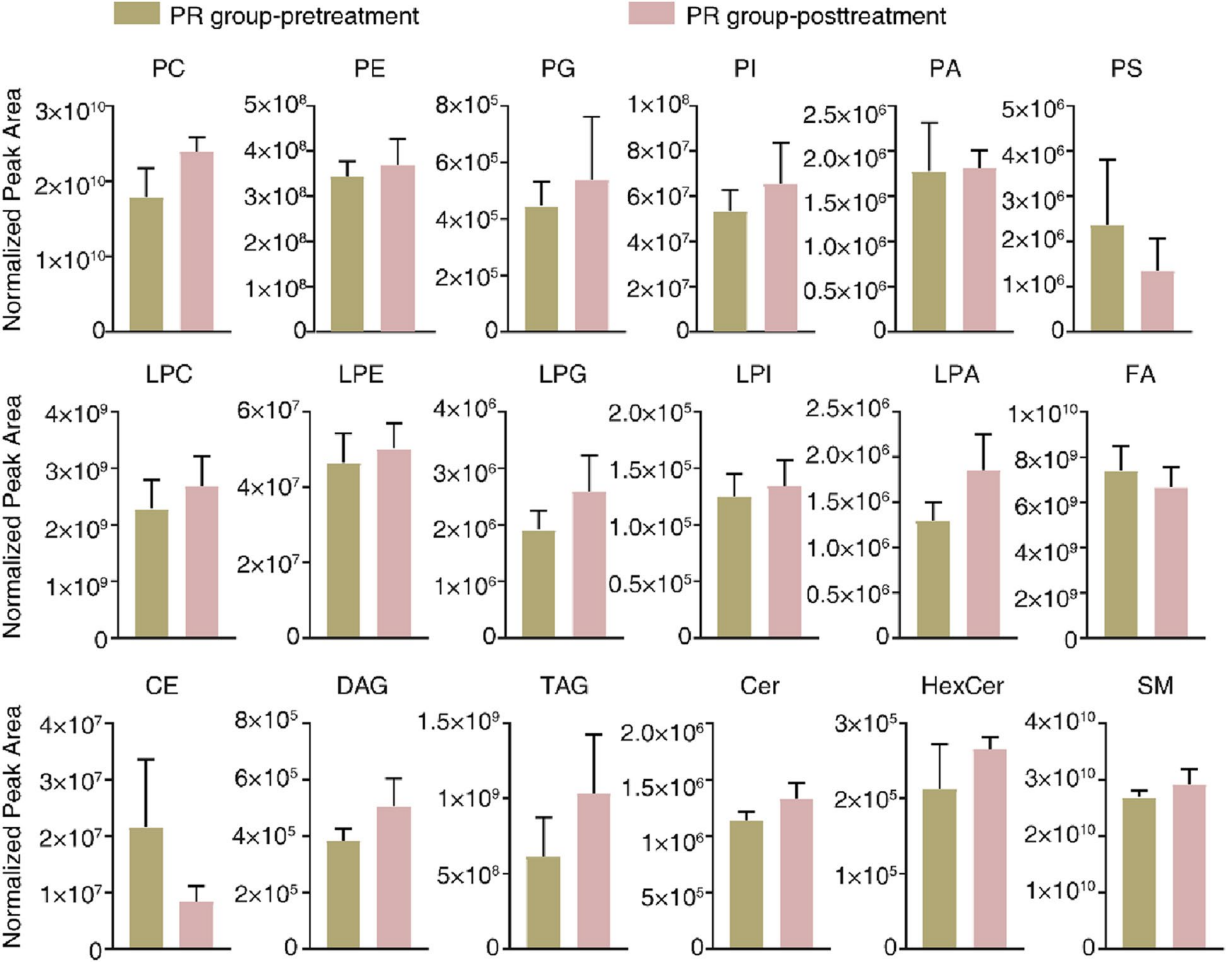
Lipid identification in advanced NSCLC patients in the PR group. In advanced NSCLC patients, lipid identification was performed on the PR group. The analysis of sample composition within the PR group involved assessing the lipid volume in each lipid category before and after administering PD-1/PD-L1 inhibitors and anlotinib treatment. Statistical significance compared to pre-treatment patients was determined using Student’s t-tests. Asterisks indicate the existence of these noteworthy disparities when *P* -value was lower than 0.05

**Fig. 3:**
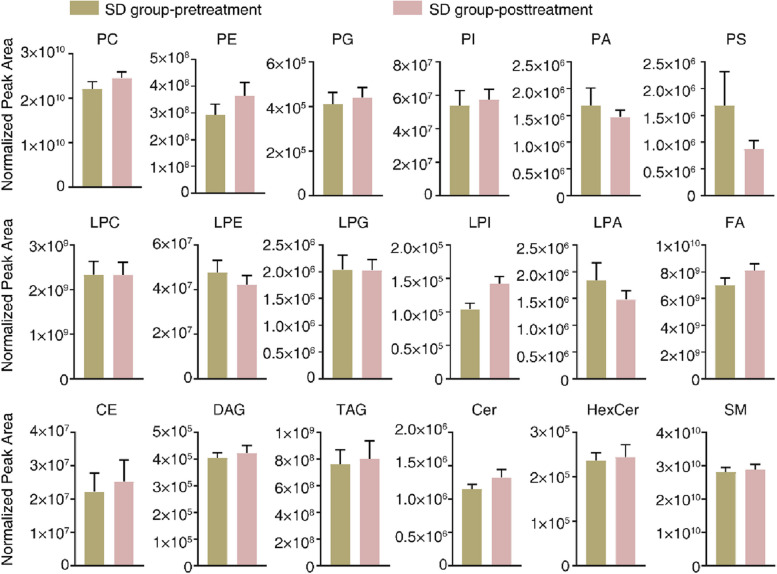
Lipid identification in advanced NSCLC patients in the SD group. The lipid composition in the SD group samples before and after administering anlotinib along with PD-1/PD-L1 inhibitor was analyzed, by assessing the lipid volume in each lipid category. Significant variations were determined using Student’s t-tests during the comparison of the patients prior to the commencement of treatment. Asterisks indicate the presence of such significant differences when *P* -value was lower than 0.05

**Fig. 4:**
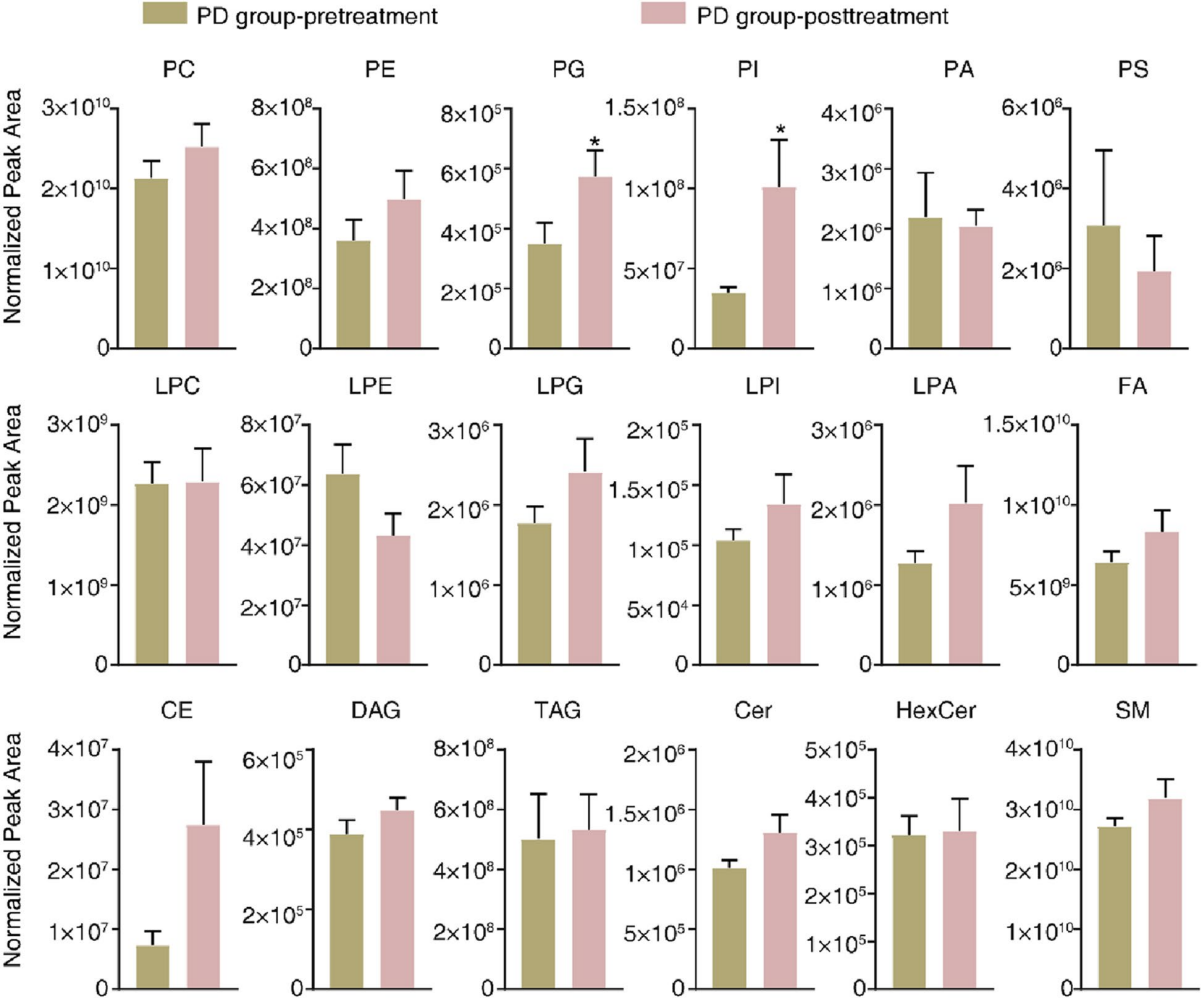
Lipid identification in advanced NSCLC patients in the PD group. After the administering anlotinib along with PD-1/PD-L1 inhibitor, the analysis of lipid volume in various lipid categories was conducted on the samples collected from the PR group. Significant variations in relation to the pre-treatment patient lipid levels were determined using Student’s t-test. Asterisks indicate the presence of such significant differences when *P* -value was lower than 0.05

The MetaboAnalyst R software package for conducting PLS-DA was employed to deploy a statistical analysis method relying on multivariate techniques in order to enhance the differentiation and detect unique metabolites among various groups. This analysis yielded evident disparities in the patients’ lipidomic profiles before (pink) and after (green) therapy, indicating a noticeable distinction among individuals from the PR, SD, and PD groups (Fig. 1A, C and E). In addition, a Pearson correlation analysis to evaluate the resemblance among various types of lipids within three distinct groups was performed (Fig. 1B, D and F). An examination of the patients’ lipidomic makeup across the three groups was conducted using lipid volume measurements. The findings revealed that there were no significant changes in lipids among the PR and SD groups between before and after treatment (Figs. 2 and 3). However, in the PD group, there was a notable increase in both PG and PI contents after treatment (Fig. 4). These results indicated that the maintenance of lipid equilibrium holds significant importance in the impressive efficacy of the conjunction of anlotinib and PD-1/PD-L1 inhibitors in treating advanced NSCLC. In addition, an imbalance in particular lipids, such as PG and PI, following treatment could suggest undesirable consequences.

### To investigate the lipid components closely associated with the therapeutic effect of PD-1/PD-L1 inhibitors in combination with anlotinib

**Fig. 5:**
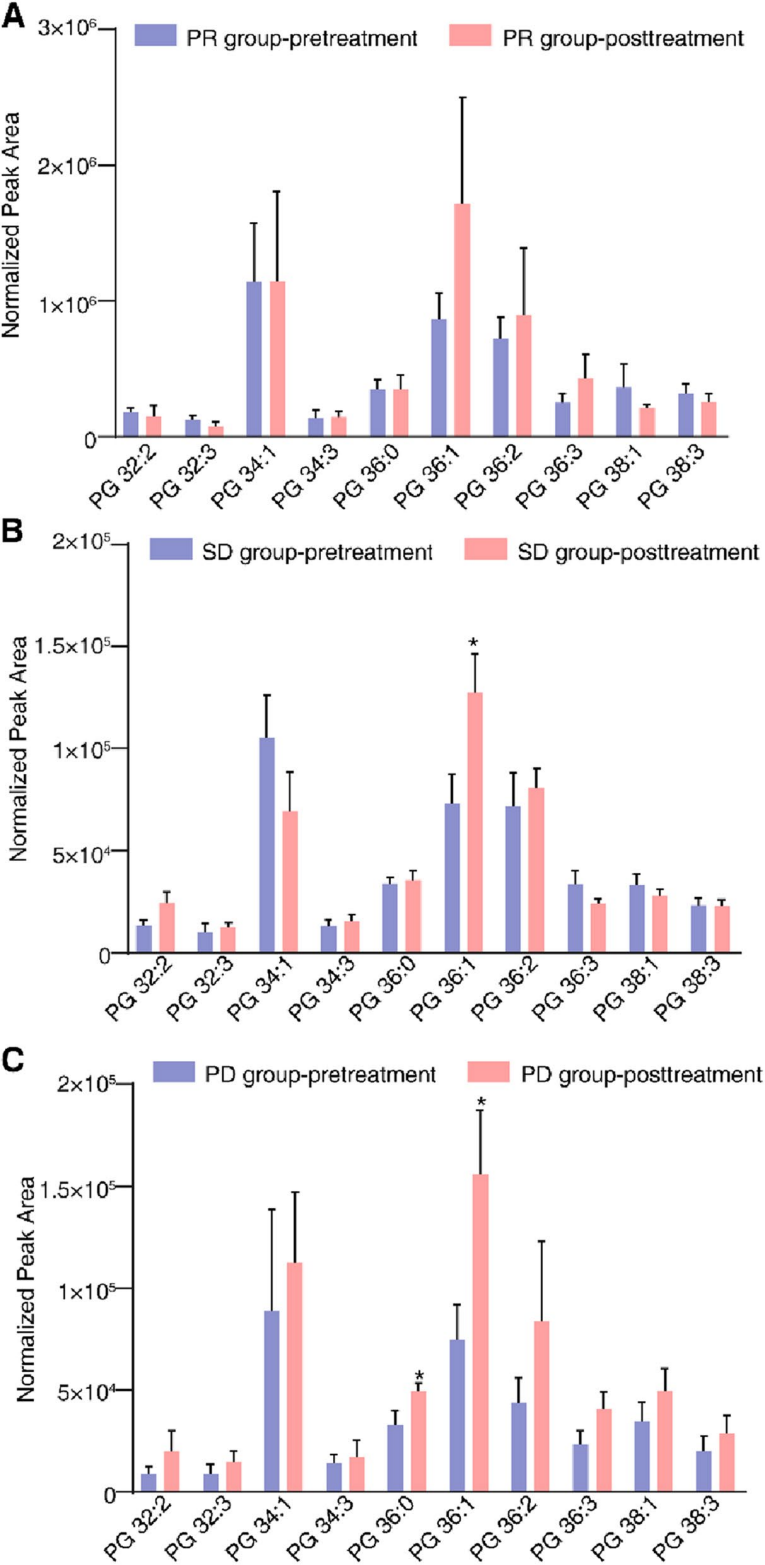
Detecting significant variations in the constituents of PG among three patient groups. **A** The PG levels in the PR group did not show any significant alteration. **B** There was a significant increase in PG 36:1 levels in the SD group after treatment. **C **PG 36:0 and PG 36:1 levels showed a significant increase in the PD group after treatment. To compare the alterations in PG levels before and after treatment, a paired two-tailed Student’s t-test was conducted within each group (PR, SD, PD). The comparison was made between the non-treatment group and the group treated with a combination of anlotinib and a PD-1 inhibitor. A *P* -value of less than 0.05 was considered statistically significant and marked with an asterisk

Specific constituents of PG and PI were systematically examined and analyzed in order to gain a deeper understanding of the lipid changes. According to the outcomes of this study, it was observed that the composition of PG exhibited a consistent upward trend. However, there were no significant changes observed in individuals of the PR group before and after treatment. Similarly, in the SD group consisting of patients with advanced NSCLC, the only notable change detected post-treatment was a significant up-regulation in the levels of PG36:1. In contrast, the PD group exhibited a substantial increase not only in PG36:1 but also in PG36:0 after treatment. Moreover, the observations revealed an overall upward trend in most PG components within the PD group following treatment, despite the lack of statistical significance in the observed differences (Fig. 5).

**Fig. 6:**
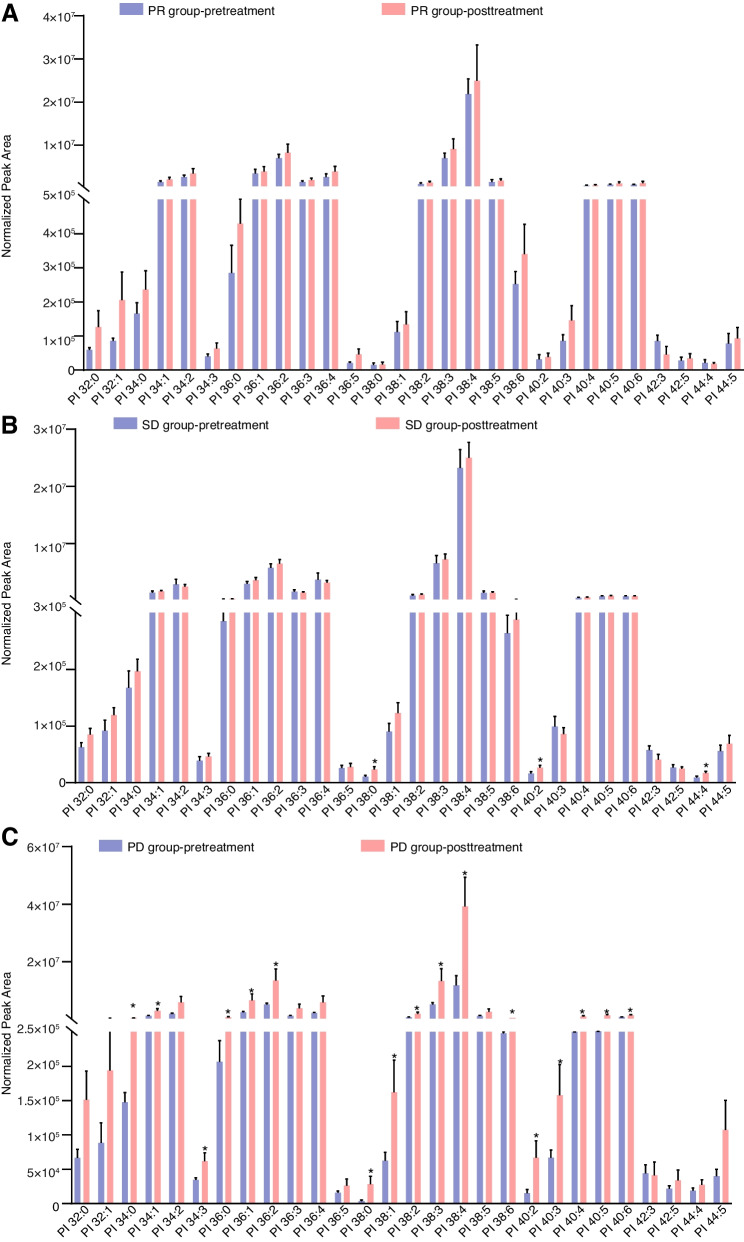
Detection of substantial alterations in the constituents of PI among three distinct groups of patients prior to and subsequent to intervention. **A **Significant alterations were not observed in the PI levels of the PR group. **B **The SD group showed a significant increase in PI 38:0, PI 40:2, and PI 44:4 levels after treatment. **C** In the PD group, more than half of the PIs exhibited a significant up-regulation after treatment. To evaluate the changes in PI levels, a paired two-tailed Student’s t-test was performed within each group (PR, SD, PD). The comparison was made between the non-treatment group and the group treated with a combination of anlotinib and a PD-1 inhibitor. Statistical significance was determined with a *P* -value of less than 0.05, which was denoted with an asterisk

After assessing the distinct elements of PI, it was observed that the composition of PI exhibited an increasing trend. However, there were no significant changes observed in individuals with advanced NSCLC from the PR group who were administered PD-1/PD-L1 inhibitors concurrently with anlotinib. A noteworthy upsurge in PI38:0, PI40:2, and PI44:4 concentrations was exhibited in advanced NSCLC patients in the SD group after therapy. Interestingly, we observed a significant change in PI levels following treatment in patients with advanced NSCLC in the PD group. After treatment, a significant up-regulation was observed in more than half of the PIs, including PI 34:0, PI 34:1, PI 34:2, PI 34:3, PI 36:0, PI 36:1, PI 36:2, PI 38:0, PI 38:1, PI 38:2, PI 38:3, PI 38:4, PI 38:6, PI 40:2, PI 40:3, PI 40:4, PI 40:5, and PI 40:6 (Fig. 6).

## Discussion

For individuals diagnosed with advanced NSCLC lacking driver mutations, the administration of anlotinib in conjunction with PD-1/PD-L1 inhibitors is considered a viable alternative treatment choice for later stages of the disease, and it has demonstrated noteworthy therapeutic efficacy [21, 22]. However, there are still patients who do not benefit from this treatment approach. Therefore, a major challenge in clinical practice is to understand why individuals respond differently to drugs. One effective strategy to solve this problem is to identify and exploit potential targets that are triggered by and downstream of cancer-causing signaling pathways. Previous investigations have shown a significant increase in the de novo synthesis of endogenous lipids in numerous cancerous cells [23–25]. Studies have demonstrated that serum metabolomic profiling can reveal metabolic alterations associated with lung cancer, including amino acids, organic acids, and nitrogen compounds [26, 27]. Additionally, lipid and lipid-like molecules have been identified as potential biomarkers for NSCLC. Lipids, as crucial components of cell membranes, undoubtedly influence the activity of proteins on the membrane [28, 29]. Membrane proteins such as epidermal growth factor receptor and tumor necrosis factor receptors play critical roles in tumor signaling pathways [30]. In this particular study, noteworthy disparities in the lipid composition among individuals with advanced NSCLC who received a combination treatment of anlotinib and PD-1/PD-L1 inhibitors were observed. The objective of this investigation was to identify lipid components that may be associated with the therapeutic efficacy of advanced NSCLC treated with a combination of anlotinib and PD-1/PD-L1 inhibitors, from a lipidomics perspective. The results derived from this research provide valuable insights into potential innovative therapeutic strategies.

During this study, the lipid compositions of individuals in the PR, PD, and SD groups were analyzed, revealing remarkable variations in the lipid composition across these groups. Further analysis identified 19 differential lipids, including two PGs and 17 PIs. PG and PI are two important classes of glycerophospholipids with diverse roles in cell signaling and lipid–protein interactions [31]. These molecules can be potential targets for novel drug development in the fight against cancer [32, 33].

As a crucial structural lipid, PG acts as a precursor of cardiolipin, which is primarily found in mitochondrial membranes, and it plays a key role in mitochondrial functionality and membrane integrity [34, 35]. Studies have shown that elevated levels of PG are present in renal cell and hepatocellular carcinomas [36, 37]. During the investigation, a notable rise in the levels of two PGs was observed among patients with advanced NSCLC belonging to the PD group who were administered PD-1/PD-L1 inhibitors alongside anlotinib. Furthermore, one PG also exhibited a significant increase in patients with advanced NSCLC in the SD group. However, no PGs that showed significant differences in patients with advanced NSCLC in the PR group. These findings suggest that the abnormal accumulation of PG after treatment could result in irreversible respiratory injury and hinder the use of alternative energy sources to glucose, ultimately leading to tumor progression [34].

PIs make up only a small portion of the phospholipid content found in cells, yet they exert a pivotal influence on the progress and development of cancer [38]. The findings in this paper indicated that more than half of the PIs showed a significant increase among individuals with advanced NSCLC in the PD group after treatment. However, only three PIs showed a significant increase in advanced NSCLC patients of the SD group after treatment, and no PIs showed significant changes in patients with advanced NSCLC in the PR group. Prior investigations established that PIs have the ability to function as building blocks for the creation of phosphatidylinositol 4,5-bisphosphate as well as phosphatidylinositol 3,4,5-trisphosphate. These substances are known to be essential in the PI3K-AKT pathway, which regulates cell survival, proliferation, invasion, and growth [39]. The observations propose that an abnormal elevation in PI concentrations following therapy could impede the advantageous effects of anlotinib combined with PD-1/PD-L1 inhibitors for patients.

To summarize, adopting a lipidomics approach, an investigation was performed that aimed at analyzing the elements that contribute to the divergent response of patients with advanced NSCLC harboring negative driver mutations when subjected to a combined therapeutic regimen of anlotinib and PD-1/PD-L1 inhibitors. Based on the results, we propose a possible mechanism by which abnormal elevations of PG and PI hinder the beneficial effects of anlotinib combined with PD-1/PD-L1 inhibitors for patients (Supplemental Fig. 1). We observed a positive correlation between PG and PI in each response group (PR, SD, and PD) during the analysis of Pearson correlation. This suggests that an increase in PG content corresponds to an increase in PI content, and vice versa. We hypothesize that the elevation of PG/PI levels could activate the PI3K-AKT pathway. This is because PI can serve as a substrate for PIP2, which is phosphorylated by PI3K. Activation of the PI3K-AKT pathway promotes tumor growth, which may explain the observed tumor progression in patients in the PD group. In these patients, the levels of PG/PI were abnormally elevated after treatment with anlotinib and a PD-1 inhibitor. However, we did not detect the activity of proteins involved in the PI3K-AKT signaling pathway. This limitation prevents us from fully supporting the proposed working model of our investigation. To validate our hypothesis, further investigations should be conducted to determine phosphoinositide 3-kinase kinase activity, as well as the concentrations of phosphoinositide (4,5) bisphosphate and phosphoinositide (3,4,5) trisphosphate. The findings provide valuable insights into lipid metabolism in advanced NSCLC, which not only offers potential for novel therapeutic approaches but also aids in the identification of new therapeutic biomarkers. In addition, lipid metabolism status has the potential to function as a prognostic indicator for determining the eligibility of patients with advanced NSCLC, who may gain advantages from the combination of anlotinib and PD-1/PD-L1 inhibitors.

### Study strengths and limitations

The main advantage of this research is to identify potential factors that may influence the therapeutic outcomes observed in advanced NSCLC patients with negative driver mutations when treated with a combination of anlotinib and PD-1/PD-L1 inhibitors. However, the investigation solely focused on changes in lipids, and the specific mechanisms through which these lipids affect advanced NSCLC treatment are still unclear. The enrolled patients included not only those with lung adenocarcinoma but also those with lung squamous cell carcinoma. It is worth mentioning that the sample size in the PD group was relatively small. Therefore, further evidence is required to comprehensively examine and understand these mechanisms.

## Conclusions and clinical perspective

The administration of anlotinib along with PD-1/PD-L1 inhibitors has demonstrated promise as a viable tactic for managing individuals with advanced NSCLC lacking driver mutations. The plausible rationale behind this lies in the capacity of the therapy to induce modifications in the lipidomics of patients with advanced NSCLC. Within this study, novel insights have been unveiled regarding the correlation between the combination of anlotinib with PD-1/PD-L1 inhibitors and distinct lipids in advanced NSCLC patients. Such discoveries propose that directing interventions toward these lipid modifications could present a hopeful and encouraging methodology for managing patients with advanced NSCLC.

### Supplementary Information

**Additional file 1: Supplemental Fig. 1.** The potential mechanism by which abnormal elevations of PG and PI hinder the beneficial effects of combining anlotinib with PD-1/PD-L1 inhibitors for patients. During the analysis of Pearson correlation, a positive correlation between PG and PI was observed in each response group (PR, SD, and PD). This indicates that an increase in PG content corresponds to an increase in PI content, and vice versa. A hypothesis is proposed that the elevation of PG/PI levels could activate the PI3K-AKT pathway. This is because PI can act as a substrate for PIP2, which is phosphorylated by PI3K. Activation of the PI3K-AKT pathway has been linked to promoting tumor growth.

## Data Availability

All data in this study can be obtained from the corresponding author up on request.
