# Supplementary material for: Lipid alterations play a role in the integration of PD-1/PD-L1 inhibitors and anlotinib for the treatment of advanced non–small-cell lung cancer
Source: Lipids Health Dis. 2024 Jan 13;23:16. doi: 10.1186/s12944-023-01960-7 (PMC10787985; doi:10.1186/s12944-023-01960-7)
Supplement: Supplementary file 1 — Additional file 1: Supplemental Fig. 1. The potential mechanism by which abnormal elevations of PG and PI hinder the beneficial effects of combining anlotinib with PD-1/PD-L1 inhibitors for patients. During the analysis of Pearson correlation, a positive correlation between PG and PI was observed in each response group (PR, SD, and PD). This indicates that an increase in PG content corresponds to an increase in PI content, and vice versa. A hypothesis is proposed that the elevation of PG/PI levels could activate the PI3K-AKT pathway. This is because PI can act as a substrate for PIP2, which is phosphorylated by PI3K. Activation of the PI3K-AKT pathway has been linked to promoting tumor growth. [file 12944_2023_1960_MOESM1_ESM.docx]

**Supplemental Figure**


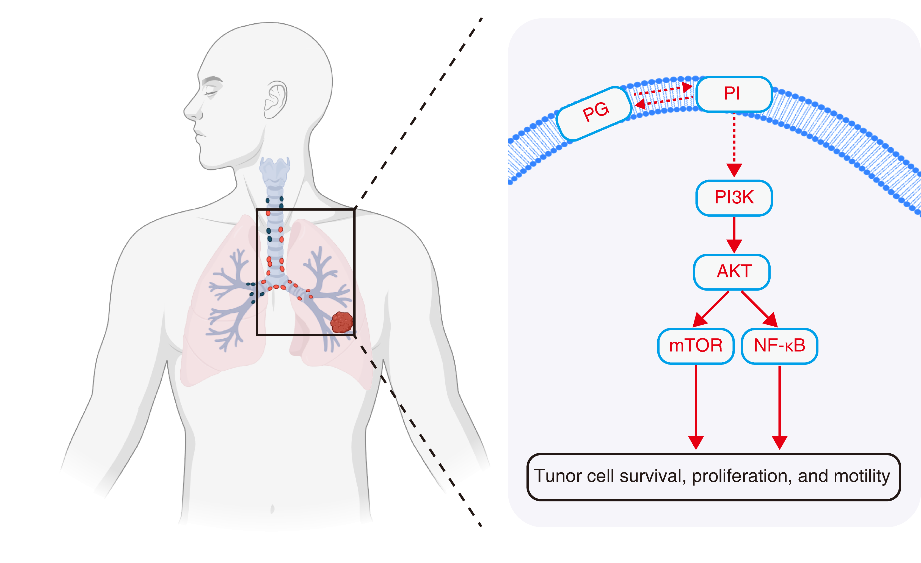


**Supplemental Figure 1 The potential mechanism by which abnormal elevations of PG and PI hinder the beneficial effects of combining anlotinib with PD-1/PD-L1 inhibitors for patients**

During the analysis of Pearson correlation, a positive correlation between PG and PI was observed in each response group (PR, SD, and PD). This indicates that an increase in PG content corresponds to an increase in PI content, and vice versa. A hypothesis is proposed that the elevation of PG/PI levels could activate the PI3K-AKT pathway. This is because PI can act as a substrate for PIP2, which is phosphorylated by PI3K. Activation of the PI3K-AKT pathway has been linked to promoting tumor growth.
